# Evaluation of the orthodontic treatment need in a paediatric sample from Southern Italy and its importance among paediatricians for improving oral health in pediatric dentistry

**DOI:** 10.4317/jced.54005

**Published:** 2017-08-01

**Authors:** Valeria Luzzi, Gaetano Ierardo, Denise Corridore, Gabriele Di Carlo, Gianni Di Giorgio, Emanuele Leonardi, Guglielmo-Giuseppe Campus, Iole Vozza, Antonella Polimeni, Maurizio Bossù

**Affiliations:** 1 Department of Oral and Maxillofacial Sciences, “Sapienza” University of Rome, Rome, Italy; 2 Istituto Nazionale di Fisica Nucleare (INFN), Sezione di Roma, Rome, Italy; 3 School of Dentistry, University of Sassari, Italy; 4 WHO Collaborating Centre for Epidemiology and Community Dentistry of Milan, University of Milan, Italy

## Abstract

**Background:**

Data from epidemiological studies investigating the prevalence and severity of malocclusions in children are of great relevance to public health programs aimed at orthodontic prevention. Previous epidemiological studies focused mainly on the adolescence age group and reported a prevalence of malocclusion with a high variability, going from 32% to 93%. Aim of our study was to assess the need for orthodontic treatment in a paediatric sample from Southern Italy in order to improve awareness among paediatricians about oral health preventive strategies in pediatric dentistry.

**Material and Methods:**

The study used the IOTN-DHC index to evaluate the need for orthodontic treatment for several malocclusions (overjet, reverse overjet, overbite, openbite, crossbite) in a sample of 579 children in the 2-9 years age range.

**Results:**

The most frequently altered occlusal parameter was the overbite (prevalence: 24.5%), while the occlusal anomaly that most frequently presented a need for orthodontic treatment was the crossbite (8.8%). The overall prevalence of need for orthodontic treatment was of 19.3%, while 49% of the sample showed one or more altered occlusal parameters. No statistically significant difference was found between males and females.

**Conclusions:**

Results from this study support the idea that the establishment of a malocclusion is a gradual process starting at an early age. Effective orthodontic prevention programs should therefore include preschool children being aware paediatricians of the importance of early first dental visit.

** Key words:**Orthodontic treatment, malocclusion, oral health, pediatric dentistry.

## Introduction

Data from epidemiological studies investigating the prevalence and severity of malocclusions in children are of great relevance to the implementation of public health programs aimed at orthodontic prevention. In this context epidemiology becomes a useful tool for the early diagnosis of malocclusions, contributing to the limitation of further aggravations of the same during the later stages of growth (1-4).

In order to evaluate the prevalence of malocclusions and the associated risk factors, several measurement indexes were defined over the years. Early examples are the Grainger’s “Treatment Priority Index” (TPI) (5), the Salzmann’s “Handicapping Malocclusion Assessment Record” (6) or the Summers’ “Occlusal Index” (7). In 1989 Brook and Shaw defined the “Index of Orthodontic Treatment Need” (IONT) with the associated “Peer Assessment” (8). This index, with the following modifications by Shaw (9), is now widely used in the clinical practice.

Epidemiological studies on the prevalence of malocclusions and on the need of orthodontic treatment (10) conducted in several countries reported a prevalence of malocclusion with a high variability, going from 32% to 93% (11-14). This inhomogeneity can be attributed to several factors: the use of different indexes, including IOTN and TPI (with several modifications), the different age, social status, and ethnic group of the samples studied, and the data collection techniques.

Aim of our study was to assess the need for orthodontic treatment in a paediatric sample from a well-defined geographical area of Southern Italy, namely the Caserta province in order to improve awareness among paediatricians about oral health preventive strategies in pediatric dentistry .While most studies in the literature focused on the adolescent age range (14,15), our study focused on the 2-9 years age group, this in order to evaluate the prevalence of malocclusions and need for treatment at an earlier age and to intercept as early as possible in childhood the clinical evolution of the malocclusion and consequently implement proper treatment protocols to prevent its aggravation.

## Material and Methods

579 children (2-9 years old) were examined in collaboration with paediatricians from the Caserta province (Italy), in the period between June 2009 and June 2010, as part of a large epidemiological study designed to establish a whole set of risk factors related to oral health.

The study protocol was conformed to the ethical guidelines of the 1975 Declaration of Helsinki and was approved by the Ethics Committee of the Policlinico “Umberto I” in Rome and the authorization to the clinical examination of the children and to the processing of the personal data has been obtained by written informed consent signed by both parents.

-Sample selection

Caserta is a southern Italian city located in the north of the Campania region. The province of Caserta and its metropolitan area have a population of about 910,000 inhabitantsa.

45 paediatricians working in the province of Caserta and belonging to the Italian Society of Preventive and Social Paediatricsb (SIPPS) participated to the project. According to the italian system, each child born in the province of Caserta is randomly assig-ned to a pediatrician belonging to the Public Health Service, of which SIPPS is a good proxy. Thus the set of children covered by SIPPS provide a representative approximation of the population in the area.

Each paediatrician recruited, on a voluntary base, a random sample of 10 to 20 children in the required age range for a routine dental check. An exclusion criterion from the study was the existence of a previous or on-going orthodontic treatment.

All patients were examined using a mirror, a disposable explorer, and a metal gauge. The children were sitting on the examination table and examiners used a portable 60W white lamp as light source.

a Data from ISTAT 2001 - 14° Censimento Generale della Popolazione e delle Abitazioni. Available on-line at http://dawinci.istat.it

b Società Italiana di Pediatria Preventiva e Sociale. Web site: http://www.sipps.it

An initial physical examination collected the family, physiological, and pathological medical histories of each subject into a medical file specifically created for the study, while the parents were required to fill a questionnaire related to non-nutritive sucking habits (use of pacifier and finger sucking) of the child from the first to sixth year of life.

Data collected for this study included information related to caries experience and its associated risk factors: this section of the study was already presented in a previous publication (16).

-Orthodontic parameters

On examination, the orthodontic parameters for overjet, reverse overjet, overbite, anterior openbite, and posterior mono- or bilateral crossbite were measured using the following criteria:

•Overjet (OVJ) – Projection of the upper central incisor on the lower one along the sagittal plane. Using a metal gauge we measured the distance between the buccal surface of the lower central incisor and the palatal surface of the upper central incisor along the parallel to the occlusal plane.

•Reverse overjet (OVJ-) – The lingual surface of the lower central incisor is positioned anterior to the buccal surface of the upper incisors. We measured the distance between the buccal surface of the upper incisors and the lingual surface of the lower incisors along the parallel to the occlusal plane.

•Overbite (OVB) – This is the overlap in the vertical plane of the upper teeth on the lower ones. The metal gauge was positioned perpendicularly to the occlusal plane and measured the section of the labial surface of the lower incisors covered by the palatal surface of the upper incisors, in the presence of rear dental contact.

•Anterior openbite (OPB) – When the upper central incisors were not in contact with the lower ones along the vertical plane, we measured the width of the resulting gap perpendicularly to the occlusal plane.

•Crossbite (CRB) – In a posterior cross-bite the buccal cusps of the upper deciduous molars and of the upper first permanent molar occlude lingually to the buccal cusps of the corresponding lower elements. Given to age range of the children, it was not possible to measure the entity of the dental discrepancy but only to identify its presence and characterize it as mono- or bilateral.

To reduce inter-examiner error, the same dental team carried out all visits. All members of the team underwent a preliminary calibration session to guarantee an unambiguous identification of the malocclusions and a coherent evaluation of the relevant occlusal parameters.

Using the measured parameters, we assigned a grade to the need for orthodontic treatment of each malocclusion following the IOTN-DHC (Index of Orthodontic Treatment Need – Dental Health Component) classification, detailed in  Table 1. Given operative constraints related to the age of the children and to the time available for the exam, some of the criteria listed in  Table 1 were modified as follow:

**Table 1:**
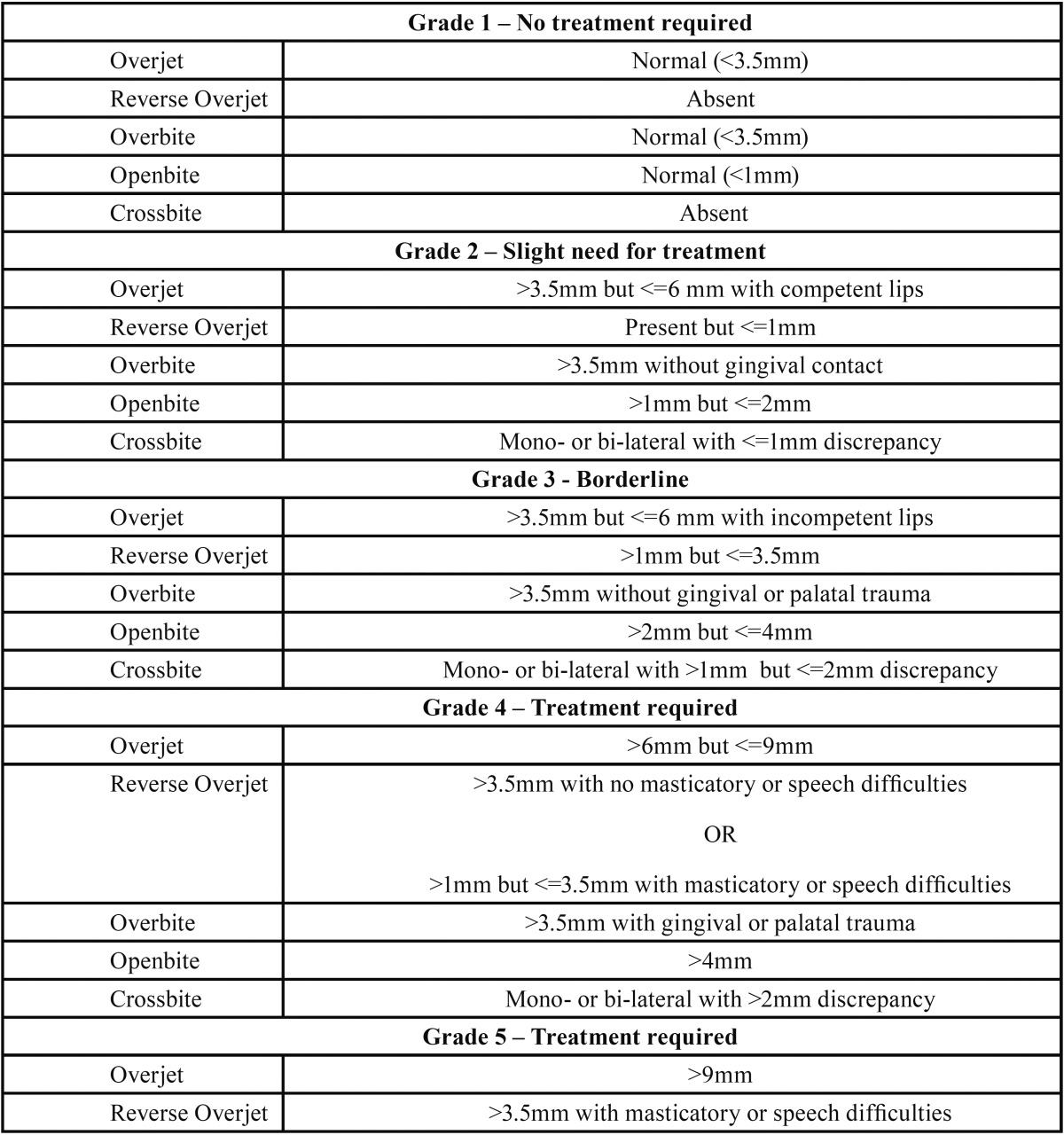
IOTN-DHC classification for the treatment need of orthodontic malocclusions.

• CRB was only reported as “present” or “absent” and, if present, it was classified as of grade 4 (i.e. to be treated).

• In presence of OVJ-, it was not possible to reliably evaluate the presence of speech and/or masticatory difficulties. This implied that it was not possible to identify grade 5 anomalies and that some of the grade 4 anomalies were classified as grade 3.

In view of these limitations, all malocclusions were then classified as “altered” if the IOTN-DHC grade was ≥ 2 and “to be treated” if the IOTN-DHC grade was ≥ 3. This choice was taken in agreement with a Swedish study of 2009 stating that IOTN-DHC grade 3 anomalies should be included in the orthodontic treatment need (17).

Finally, we assigned to each subject an overall IOTN-DHC grade, defined as the grade corresponding to the severest occlusal anomaly.

-Statistical methods

Prevalence was evaluated using the Wilson method with a 95% confidence interval.

The chi-square test of Pearson was used to compare the orthodontic treatment need in both sexes. A *p* value <0.05 was considered statistically significant.

All calculations were performed using the SPSS 13.0 software (SPSS Inc., Chicago, Illinois) and the CIA Software 2.0.

## Results

The sample included a total of 579 children (306 males and 273 female) ranging in age from 2 to 9 years (mean age 5.73, median 6, SD 1.65). Almost all children were of Italian nationality (99.3%). Due to lack of compliance during orthodontic examination, in 20 subjects it was not possible to reliably measure the IOTN-DHC. These subjects were therefore excluded from the study.

For each malocclusion and for the overall need for orthodontic treatment we evaluated the distribution of IOTN-DHC grade both in the full sample and in the males and females sub-samples. Using the Wilson method we then evaluated the prevalence of each malocclusion in terms of the presence of an alteration (sum of IOTN-DHC grades from 2 to 5) and in terms of the need for treatment (sum of IOTN-DHC grades from 3 to 5). Finally, we used the chi-square test to assess the likelihood that this prevalence is different between males and females. The results for each malocclusion (OVJ, OVJ-, OVB, OPB, and CRB) together with the overall IOTN-DHC grade are summarized in  Table 2.

**Table 2:**
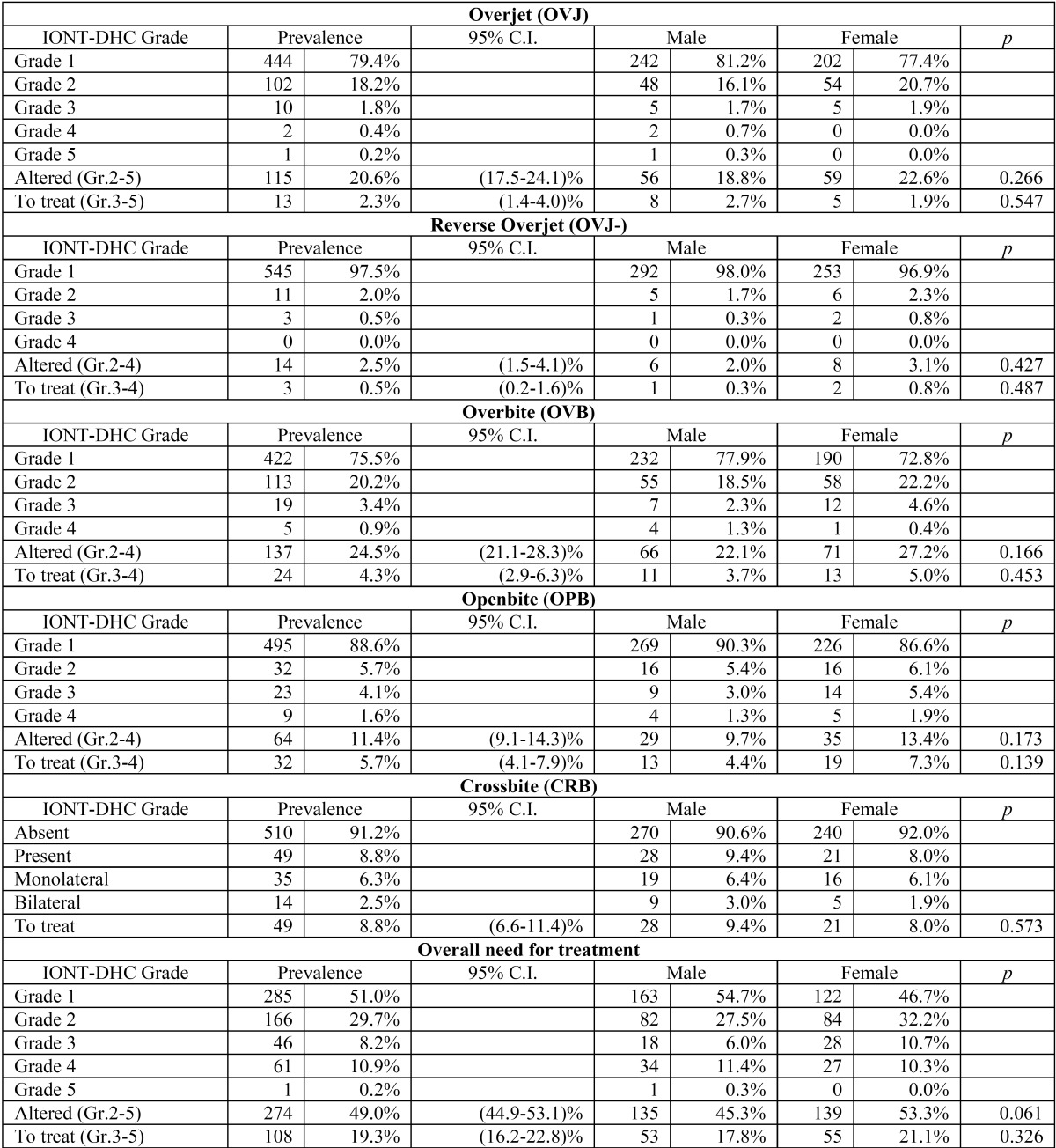
Prevalence of the IOTN-DHC grade for Overjet (OVJ), Reverse Overjet (OVJ-), Overbite (OVB), Openbite (OPB), Crossbite (CRB) and for the overall need for treatment. A comparison between male and female subjects is also shown.

**Figure 1:**
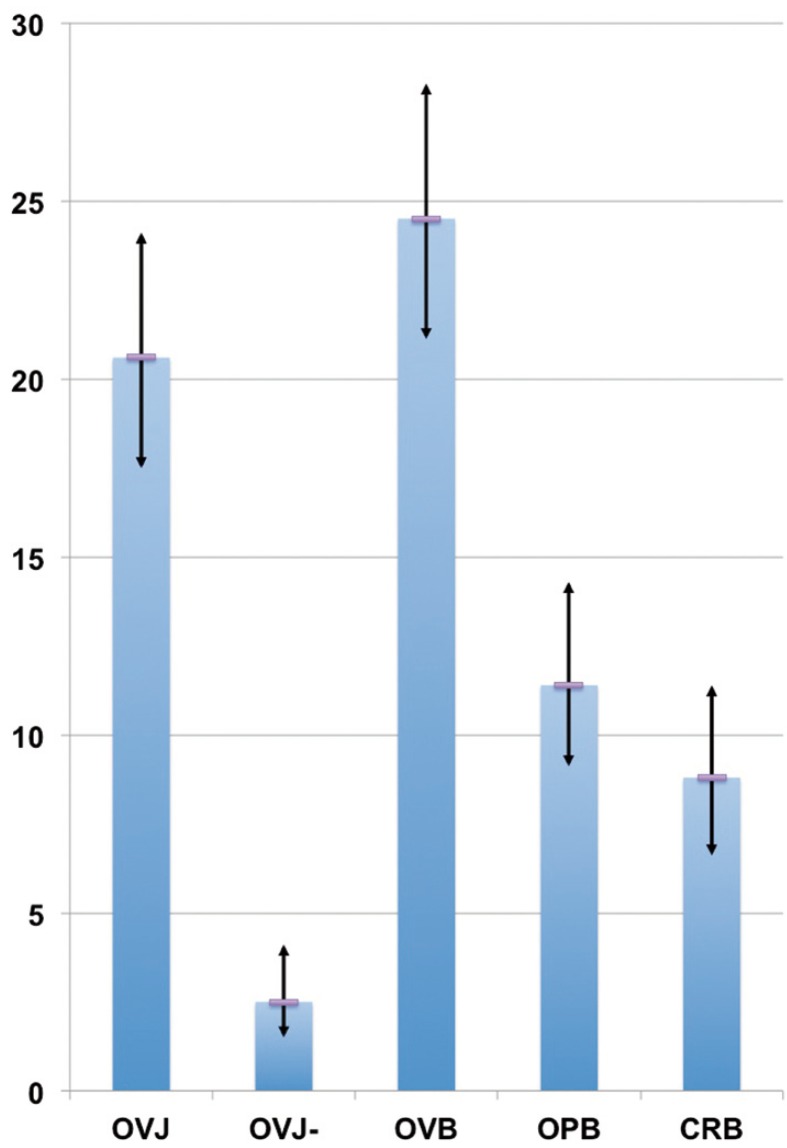
Prevalence of altered occlusal parameters for overjet (OVJ), reverse overjet (OVJ-), overbite (OVB), openbite (OPB), and crossbite (CRB) in our sample. Vertical bars indicate the 95% C.I.

From these results we observe that the most frequently altered occlusal parameter is the overbite (prevalence: 24.5%, 95% C.I.: (21.1-28.3)%) followed by overjet (20.6%, (17.5-24.1)%), openbite (11.4%, (9.1-14.3)%), crossbite (8.8%, (6.6-11.4)%) and reverse overjet (2.5%, (1.5-4.1)%), as shown in Figure 1, with an overall prevalence of 49.0% and a 95% C.I. of (44.9-53.1)%.

**Figure 2:**
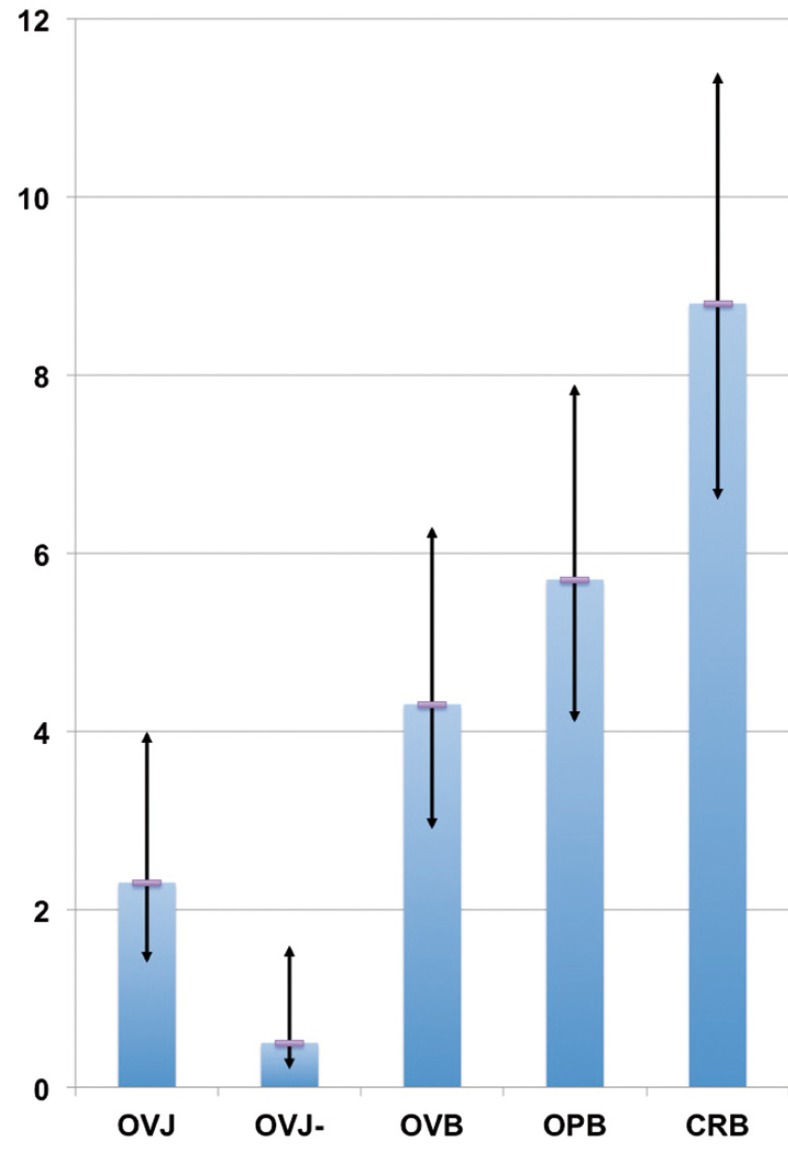
Prevalence of occlusal anomalies with need for orthodontic treatment for overjet (OVJ), reverse overjet (OVJ-), overbite (OVB), openbite (OPB), and crossbite (CRB) in our sample. Vertical bars indicate the 95% C.I.

On the other hand, the occlusal anomaly that most frequently presents a need for orthodontic treatment is the crossbite (prevalence: 8.8%, 95% C.I.: (6.6-11.4)%) followed by openbite (5.7%, (4.1-7.9)%), overbite (4.3%, (2.9-6.3)%), overjet (2.3%, (1.4-4.0)%) and reverse overjet (0.5%, (0.2-1.6)%), as shown in Figure 2, with an overall prevalence of 19.3% and a 95% C.I. of (16.2-22.8)%.

None of the malocclusions presented a statistically significant difference between males and females.

## Discussion

The primary objective of this study was to analyse the need for orthodontic treatment in a paediatric sample from the Caserta province in order to acquire elements for the implementation of Public Health protocols related to malocclusions prevention. The need for orthodontic treatment was evaluated using the IOTN-DHC index.

We observed the highest prevalence of need for orthodontic treatment for the crossbite (9.0 ± 2.4%) followed by openbite (6.0 ± 1.9%). These data are in agreement with those reported in the literature, which always identified the crossbite as having a priority in the need for treatment since the early childhood (18-20).

When evaluating the results for the overall IONT-DHC grade, we note that two earlier studies used this method to examine paediatric populations of Southern Italy. In 2007, Nobile *et al.* (21) studied a sample of children in the 11-15 years age range from middle and high schools of Catanzaro and found a high prevalence of malocclusion: approximately 59.5% of the sample required orthodontic treatment of 4th and 5th grade IONT. In 2009 another study (22) analysed a sample of 703 12-year-old children from several junior high schools in Naples, and found that 27.3% of the sample required orthodontic treatment of 4th and 5th IONT-DHC grade. The first French study using the IONT dates back to 2006: it examined 511 children in the 9-12 years age range and reported a prevalence of 21.3% of orthodontic treatment need (23). This finding agrees with a Spanish study of 2009 reporting a need for treatment equal to 21.8% in a group of 12-year-old children (24). This prevalence is comparable to that found in other studies that used the IONT index8, (25-28), but lower than that observed, for example, in Africa (29,30) or in Turkey (31), and Jordan (32) in which the prevalence of malocclusion that definitely needs a treatment varies between 34% and 83.2%.

Our research shows that the prevalence of children who definitely need an orthodontic treatment plan (IOTN-DHC grades 4 and 5) is of 11.1% and rises to 19.3% if we include grade 3. These figures are lower than the cited studies: this is probably due to the fact our sample was aged between 2 and 9 years, i.e. lower than the average of other studies. On the other hand our analysis shows that about 49% of children present at least one occlusal anomaly. This figure, combined with the above considerations on the need for treatment, indicates that the establishment of a malocclusion is a gradual process starting at an early age. Any effective orthodontic prevention program should therefore include preschool children in order to intervene before malocclusions stabilize or worsen at a later age. The results obtained from this study point out that pediatricians must know and be aware about dental health in order to detect the principal and more frequent problems and send the pediatric patients to orthodontists and pediatric dentists (33,34). Lack of knowledge and awareness on information, health education and health promotion can potentially increase the oral health risk status of children as stated by international and national Oral Health guidelines (35,36).
